# Brain‐Machine Interfaces for Dementia: A Novel Approach to Early Detection and Personalized Rehabilitation

**DOI:** 10.1002/alz70855_106434

**Published:** 2025-12-25

**Authors:** Sifat R Wahid, Arshia A Khan

**Affiliations:** ^1^ University of Minnesota Duluth, Duluth, MN, USA

## Abstract

**Background:**

Dementia, a syndrome characterized by progressive cognitive decline, affects over 55 million people globally, with Alzheimer's disease accounting for 60–70% of cases. Traditional interventions, such as medication and cognitive therapies, have limited success in slowing down dementia progress. In this abstract, we propose Brain‐Machine Interfaces (BMIs), which enable direct communication between the brain and external devices, present a novel opportunity to detect early stage dementia and help in rehabilitation of dementia patients. BMIs can detect early neural biomarkers of cognitive decline, such as altered theta‐gamma oscillations or diminished functional connectivity, while offering real‐time therapeutic interventions like neurofeedback. This exploratory study investigates the feasibility of non‐invasive BMIs as tools for monitoring cognitive health and enhancing resilience in dementia patients, focusing on usability, neural correlates of decline, and patient engagement.

**Methods:**

Proposed Framework for Detection:

BMIs could use non‐invasive sensors (e.g., EEG, fNIRS) to monitor biomarkers linked to early dementia, such as:

Theta‐gamma phase‐amplitude coupling: Reduced coupling correlates with memory deficits.

Event‐related potentials (ERPs): Delayed P300 responses during attention tasks could indicate processing speed decline.

Machine learning algorithms can be employed to analyze these patterns to classify dementia risk before clinical symptoms show.

Passive Monitoring:Wearable BMIs could track neural activity during daily activities (e.g., reading, social interactions) to detect anomalies in real time. For example, erratic frontal alpha oscillations during problem‐solving might signal executive dysfunction.

Proposed Framework for Rehabilitation:

BMIs could provide real‐time feedback during cognitive exercises. For instance:

A memory game adjusts difficulty based on hippocampal theta activity.

Personalized Engagement:

Gamified interfaces, tailored to user preferences (e.g., music, visual themes), could improve adherence.

**Results:**

Detecting dementia using EEG and machine learning classifiers have shown promising results. For example, Joshi et al. used EEG signals and a BiLSTM model which achieved an accuracy of 97.27%.

**Conclusion:**

This exploratory study proposes that BMIs hold transformative potential for dementia care, bridging early detection with dynamic, personalized rehabilitation. By utilizing neural biomarkers, BMIs could identify at‐risk individuals years before symptom onset, while adaptive neurofeedback systems might slow decline by strengthening cognitive reserve.

